# Motivational and demotivational teaching styles in physical education: the role of basic psychological needs

**DOI:** 10.3389/fpsyg.2026.1839188

**Published:** 2026-06-09

**Authors:** Ying Li, Naiming Fu

**Affiliations:** 1 Finance Department, Shandong Sport University, Jinan, Shandong, China; 2 Shandong Sport University, Jinan, Shandong, China

**Keywords:** basic psychological needs, demotivational teaching style, mediation analysis, motivational teaching style, physical education, Self-Determination Theory

## Abstract

This study investigated the associations between students' perceived motivational and demotivational teaching styles and motivation in physical education (PE), with basic psychological needs (autonomy, competence, and relatedness) examined as mediators within the Self-Determination Theory framework. A total of 412 secondary school students (aged 13–16 years) completed a cross-sectional survey using validated instruments assessing teaching-style perceptions, psychological need satisfaction, and overall motivational orientation in PE. Structural equation modeling (SEM) and bootstrapped mediation analyses (5,000 resamples) were conducted to test the hypothesized direct and indirect relationships. Results indicated that perceived motivational teaching style was positively associated with autonomy, competence, and relatedness, whereas demotivational teaching style was negatively associated with all three needs. Moreover, the three psychological needs statistically mediated the links between teaching styles and overall motivational orientation, with partial mediation observed for both supportive and controlling instructional behaviors. Among the mediators, relatedness showed the strongest indirect link for motivational teaching style, while autonomy appeared most sensitive to demotivational teaching style. These findings suggest that psychological needs may serve as important statistical pathways linking students' perceptions of teacher behavior with motivation in PE. The study contributes to SDT-based PE literature by simultaneously examining supportive and controlling instructional styles within an associational framework. Practical implications highlight the importance of teacher training programs that promote autonomy-supportive strategies and minimize controlling behaviors to support more engaging, need-supportive, and motivationally adaptive PE environments.

## Highlights

- Motivational teaching styles positively influence students' autonomy, competence, and relatedness in physical education.
- Demotivational or controlling teaching styles negatively affect students' basic psychological needs and motivation.
- Basic psychological needs mediate the relationship between teaching styles and students' motivation, confirming Self-Determination Theory mechanisms.
- The study simultaneously examines both supportive and controlling instructional behaviors, addressing a gap in PE literature.
- Findings provide practical guidance for teacher training programs to foster need-supportive learning environments that enhance student engagement and wellbeing.

## Research background

Physical education (PE) plays a critical role in shaping students' lifelong engagement in physical activity, health behavior, and psychosocial development (Martín-rodríguez and Madrigal-Cerezo, 2025). Beyond developing physical skills, contemporary PE also aims to foster students' motivation, autonomy, and positive attitudes toward movement and exercise (Hutmacher et al., 2020). However, many students experience declining motivation during their school years, which is associated with lower long-term participation in physical activity (Antunes et al., 2024; Choi et al., 2024). It is not solely due to reduced interest in exercise; rather, it reflects the interaction between students' psychological experiences and the instructional climate in PE lessons. Controlling teaching practices, excessive performance evaluation, repetitive tasks, limited student choice, and repeated experiences of competence frustration are linked with lower enjoyment, perceived agency, and willingness to engage in PE. Understanding how classroom-based pedagogical practices relate to students' psychological engagement is critical for both sport pedagogy and sport psychology research (Su and Liu, 2025).

One theoretical framework widely used to explain motivational processes in educational and sport contexts is Self-Determination Theory (SDT) (Li J. et al., 2025). Although other motivational perspectives, such as Achievement Goal Theory and Social Cognitive Theory, have provided valuable insights into students' goal orientations, perceived competence, and self-efficacy in PE, SDT is particularly suitable for the present study. It explains how social and instructional environments are associated with students' basic psychological needs and, consequently, the quality of their motivation. SDT posits that human motivation is strongly shaped by the satisfaction or frustration of three basic psychological needs: autonomy, competence, and relatedness (Hernández and Martínez, 2025). When these needs are supported within a learning environment, students tend to show more self-determined forms of motivation. These forms of motivation are linked to higher engagement, persistence, and wellbeing (Li Y. et al., 2025). Conversely, when these needs are not supported, students may experience controlled motivation, amotivation, or negative emotional outcomes (Evans et al., 2024). In the context of PE, teachers play a central role in students' motivational experiences. Their instructional behaviors are associated with students' perceptions of autonomy support, competence feedback, and social connectedness during learning activities (Guo et al., 2025).

In recent years, increasing attention has been given to teaching styles that either support or hinder students' psychological needs. Motivational teaching styles, including autonomy-supportive instruction, constructive feedback, and inclusive interaction, are associated with higher levels of students' intrinsic motivation, enjoyment, and participation in physical activity (Carriedo et al., 2023). In contrast, demotivational teaching styles, such as controlling behaviors, excessive criticism, neglect of student perspectives, or psychologically pressuring instruction, are linked to higher levels of psychological need frustration, lower motivation, and reduced engagement in PE classes (García-cazorla et al., 2026). The conceptual distinction between motivational and demotivational teaching styles requires careful clarification. These two teaching styles should not be treated merely as opposite ends of a single continuum. Supportive teaching primarily facilitates need satisfaction, whereas demotivational or controlling teaching is associated with need frustration, pressure, and disengagement. In this sense, motivational and demotivational teaching styles represent distinct pedagogical dimensions with potentially asymmetrical psychological consequences for students' motivational experiences in PE settings (Ruos et al., 2025). Therefore, it is important to distinguish teaching behaviors that support students' psychological needs from those that are linked to need frustration, rather than assuming that the absence of motivational teaching automatically indicates demotivational teaching.

Despite the growing body of literature examining motivation in physical education, several issues still warrant further empirical attention. First, many previous studies have primarily focused on motivational teaching behaviors, particularly autonomy-supportive strategies. Relatively fewer studies have examined demotivational teaching styles and their associations with students' psychological experiences in PE contexts (Sergio et al., 2025). Second, existing SDT-based studies have strongly established that supportive teaching behaviors are beneficial for students' motivation. Thus, the present issue is not whether need-supportive instruction is positive. Rather, it concerns whether supportive and demotivational teaching styles show similar or different associations with psychological need pathways when examined within the same pedagogical model (Dammert et al., 2025). Third, prior research has often examined teaching behaviors and motivational outcomes through direct associations. Less comparative attention has been given to whether autonomy, competence, and relatedness differ in their statistical mediating roles across supportive and demotivational instructional conditions. This creates a more specific conceptual limitation. The literature has not fully clarified whether motivational and demotivational teaching styles are associated with parallel patterns of need satisfaction or with differentiated need-based pathways related to students' motivation (Tilga et al., 2023).

Although previous studies grounded in Self-Determination Theory have extensively examined autonomy-supportive teaching behaviors in physical education, less attention has been given to the comparative examination of motivational and demotivational teaching styles within the same analytical model. Most existing studies tend to investigate supportive instructional behaviors independently. As a result, they provide less insight into how controlling or demotivational teaching practices are associated with students' psychological experiences when considered alongside motivational teaching practices in PE settings. Furthermore, limited research has explored whether autonomy, competence, and relatedness differ in their statistical mediating roles when supportive and demotivational teaching styles are examined together. Therefore, the present study offers a comparative extension of existing SDT-based research by examining both supportive and undermining instructional approaches within one structural model. It also explores the differentiated mediating functions of basic psychological needs in school-based physical education, without claiming to introduce a fundamentally new theoretical pathway.

Furthermore, empirical evidence remains limited regarding whether autonomy, competence, and relatedness play different mediating roles in the associations between contrasting teaching styles and students' motivational responses in PE (Behzadnia et al., 2025). Examining these pathways may clarify how specific teaching practices are related to students' psychological engagement and participation in PE (Franco et al., 2025). From a pedagogical perspective, such evidence may help teachers strengthen autonomy-supportive and inclusive practices while reducing controlling behaviors associated with lower need satisfaction. Therefore, this study examines the associations between perceived motivational and demotivational teaching styles and students' motivational experiences in PE, with autonomy, competence, and relatedness tested as statistical mediators. Rather than proposing a new SDT pathway, the study provides additional comparative evidence on how these psychological needs function within an established SDT-based model. The findings may offer practical guidance for creating learning environments associated with more sustained engagement in physical activity.

### Study design

This study employed a quantitative cross-sectional survey design to examine the associations among students' perceived motivational and demotivational teaching styles and students' basic psychological need satisfaction in physical education. The study was theoretically grounded in Self-Determination Theory (SDT), which posits that the satisfaction of autonomy, competence, and relatedness plays a central role in shaping individuals' motivation and engagement in learning contexts. A survey-based approach was adopted because the research aimed to capture students' subjective perceptions of teaching behaviors and their psychological experiences in authentic physical education settings. These perceptions are considered critical indicators of motivational processes in educational environments. Accordingly, motivational and demotivational teaching styles in this study were operationalized as students' perceived teaching behaviors rather than as objectively observed instructional practices. This approach is consistent with SDT-based educational research, which emphasizes students' subjective experiences of the learning environment as proximal correlates of psychological need satisfaction and motivation.

Several procedural precautions were applied to reduce potential response bias and common method variance. Participants were informed that their responses would be anonymous and confidential, that there were no right or wrong answers, and that their participation would not affect their PE grades or relationship with their teacher. The questionnaire was organized into separate sections for different constructs, and validated instruments with contextually adapted wording were used to improve clarity and reduce ambiguity. However, because all major constructs were measured using students' self-reported perceptions at a single point in time, the design may still be vulnerable to subjective response bias and common method variance. Therefore, the findings should be interpreted as reflecting students' perceived teaching environment rather than objectively verified teacher behavior.

The study tested a theoretical model in which students' perceived motivational teaching styles and demotivational teaching styles were hypothesized to be associated with the satisfaction of students' basic psychological needs. The proposed model was theoretically guided and explanatory in orientation, rather than deterministic. Although the cross-sectional design does not allow for causal inference, it enables the examination of theoretically grounded structural relationships among psychological and pedagogical variables within a natural classroom context. Therefore, all relationships examined in this study should be interpreted as associational rather than causal. The proposed model was specified based on SDT; however, the cross-sectional design does not allow reciprocal, temporal, or dynamic relationships among teaching styles, psychological needs, and motivation to be examined.

The unit of analysis in this study was the individual student, as the primary focus was on students' perceptions of their teachers' instructional behaviors and the corresponding psychological experiences during physical education classes. Nevertheless, students were nested within PE classes and teachers, indicating a hierarchical data structure. Multilevel modeling was not applied because the study was designed primarily to examine individual-level perceptual associations, and teacher- or class-level variables were collected only as contextual information rather than as higher-level predictors. In addition, the number of higher-level units, particularly PE classes and teachers, was limited for estimating a stable multilevel structural model. This nested structure was therefore not explicitly modeled in the present analysis; as a result, potential dependency among students from the same class or teacher may have affected standard errors, confidence intervals, and significance tests. Future research should consider multilevel modeling, multilevel structural equation modeling, or cluster-adjusted standard errors to account for classroom- and teacher-level effects. Longitudinal and multilevel designs are also needed to examine reciprocal and dynamic processes between perceived teaching behaviors, psychological need satisfaction, and students' autonomous motivational orientation over time.

### Participants

Participants in this study were secondary school students enrolled in compulsory physical education (PE) classes. A multi-stage cluster sampling procedure was used to recruit participants from several schools. However, because the participating schools were selected based on institutional permission and accessibility to the research team, the school selection process should be understood as convenience-based rather than fully representative of all secondary schools. First, five secondary schools were selected based on institutional permission and accessibility. Within each participating school, intact PE classes were randomly selected to participate in the study. All students within the selected classes were invited to complete the questionnaire. Therefore, while the use of intact classes allowed data to be collected in authentic PE settings, the convenience-based selection of schools may limit the external validity and generalizability of the findings to broader educational contexts.

A total of 452 students were initially invited to participate in the study. After screening for incomplete responses and patterned answering, 412 valid questionnaires were retained for the final analysis, resulting in a response rate of 91.2%. Incomplete responses were excluded when questionnaires contained substantial missing data, particularly when respondents failed to complete one or more main scale sections. Patterned answering was identified when respondents selected the same response option across most questionnaire items, showed repetitive response sequences across different sections, or provided responses that suggested insufficient engagement with the questionnaire. These screening procedures were applied before the main statistical analyses to improve data quality and reduce the influence of invalid response patterns. The final sample consisted of 412 students, including 206 males and 206 females, aged 14–17 years (*M* = 15.48, SD = 0.92).

The participants were drawn from 18 physical education classes taught by 10 certified PE teachers with teaching experience ranging from 5 to 18 years. Teacher-related information was reported to describe the instructional context of the participating classes. However, teacher-level variables were not incorporated into the analytical model because the study was designed to examine individual-level student perceptions of teaching behaviors and psychological need satisfaction. In addition, the number of higher-level units, particularly 18 PE classes and 10 PE teachers, was limited for estimating a stable multilevel structural model. Students attended physical education classes 2–3 times per week, with each session lasting approximately 45–60 min, which is consistent with the standard curriculum requirements for secondary school physical education. All participants were actively engaged in structured PE programs that included a variety of physical activities, such as team sports, individual skill development, and fitness training.

The inclusion criteria required students to be currently enrolled in compulsory PE classes, present during the data collection period, and willing to participate in the study. Students were excluded if they did not complete the questionnaire adequately, provided patterned responses, or were unable to provide valid questionnaire data for the main study variables. The final sample size was considered adequate for structural equation modeling (SEM) analysis. Specifically, the sample exceeded the commonly recommended minimum of 200 participants for SEM and provided a sufficient participant-to-parameter ratio for estimating the proposed latent variable model. Nevertheless, because students were nested within PE classes and teachers, the assumption of fully independent observations may not have been completely satisfied. This hierarchical structure was not modeled in the present analysis and is therefore acknowledged as a methodological limitation, particularly in relation to parameter estimation, standard errors, and generalizability.

## Ethical considerations

The study was conducted in accordance with the ethical principles outlined in the Declaration of Helsinki for research involving human participants. Permission to conduct the research was obtained from the participating schools and physical education teachers prior to data collection. Because the participants were minors, written informed consent was obtained from both students and their parents or legal guardians before participation in the study. Participants were informed about the purpose of the research, the voluntary nature of their participation, and their right to withdraw from the study at any time without any academic consequences. To ensure confidentiality and anonymity, students were instructed not to provide any identifying information on the questionnaires. Data collection was conducted in a classroom setting under the supervision of the researchers. Physical education teachers were not involved in the administration of the questionnaires to minimize potential response bias and perceived pressure on students. All collected data were treated confidentially and used solely for research purposes. The data were stored securely and were accessible only to the research team.

### Instruments

#### Motivational teaching style

Students' perceptions of motivational teaching style were assessed using an adapted version of the Learning Climate Questionnaire (LCQ) developed by Johnmarshall Reeve and Edward L. Deci. The LCQ is widely used to measure autonomy-supportive teaching behaviors within the framework of Self-Determination Theory. The scale captures instructional behaviors that are associated with students' psychological need satisfaction for autonomy, competence, and relatedness in educational settings, particularly in physical education contexts. In the present study, the LCQ was adapted to the physical education context by modifying item wording to refer specifically to PE teachers and PE learning activities. For example, general references to “teacher” or “instructor” were adjusted to “PE teacher,” and classroom-related wording was revised to reflect physical activity, skill practice, and PE lesson situations.

The adaptation process followed several steps to ensure contextual relevance and content clarity. First, the original items were reviewed by the research team to identify wording that required modification for the PE setting. Second, the adapted items were evaluated by experts in physical education, sport pedagogy, and educational psychology to assess content validity, conceptual consistency, and appropriateness for secondary school students. The expert review focused on whether the adapted items retained the theoretical meaning of autonomy-supportive teaching while being suitable for PE lessons and understandable for the target age group. Third, minor wording adjustments were made to improve clarity and readability without changing the theoretical meaning of the original items. Where translation was required, the items were translated and checked to ensure semantic equivalence with the original scale. The translation check focused on preserving the conceptual meaning of the original items rather than producing literal word-for-word equivalence. A preliminary check was also conducted to ensure that students could understand the adapted items before the main data collection. This preliminary check helped identify unclear wording and confirm that the items were contextually appropriate for secondary school PE classes.

The adapted instrument consisted of 12 items designed to assess students' perceptions of their physical education teachers' motivational instructional practices. Students were asked to indicate the extent to which each statement reflected their teacher's behavior during PE classes. Responses were recorded using a five-point Likert scale ranging from 1 (strongly disagree) to 5 (strongly agree), with higher scores indicating stronger perceptions of motivational teaching behaviors. Example items include: “My PE teacher provides us with choices during activities,” and “My PE teacher encourages us to develop our own ways of performing tasks.” Previous studies have demonstrated that the LCQ exhibits strong psychometric properties across educational and sport contexts. In the present study, the scale demonstrated good internal consistency, with a Cronbach's alpha coefficient exceeding the recommended threshold of 0.80, indicating satisfactory reliability.

#### Demotivational teaching style

Students' perceptions of demotivational teaching style were measured using an adapted version of the Controlling Coach Behaviors Scale (CCBS) developed by Christopher Bartholomew and colleagues. The CCBS has been widely applied in sport and physical education research to assess controlling and psychologically pressuring instructional behaviors. Within the perspective of Self-Determination Theory, controlling instructional practices are considered important because they are theoretically associated with lower psychological need satisfaction and less self-determined motivation. They may also be conceptually linked to need frustration, although need frustration was not directly measured in the present study. Because the original CCBS was developed primarily in sport coaching contexts, the items were adapted to reflect the instructional role of PE teachers rather than coaches. References to “coach” were modified to “PE teacher,” and examples of controlling behaviors were adjusted to fit PE classroom situations, such as teacher pressure during skill practice, criticism during performance tasks, and controlling communication during class activities.

The adaptation of the CCBS followed the same procedure used for the LCQ. The research team first reviewed all items for conceptual relevance to PE lessons. Expert reviewers then examined whether the adapted items adequately represented controlling or demotivational teaching behaviors in school-based physical education. Particular attention was given to preserving the theoretical meaning of controlling instruction while ensuring that the wording was understandable for secondary school students. The expert review also considered whether the adapted items reflected realistic PE classroom situations, including teacher pressure during performance tasks, public correction, and controlling communication during skill practice. Minor linguistic modifications were made to improve contextual fit, but the core meaning of the original construct was retained. Where wording was adapted from coaching to PE teaching, the emphasis was placed on contextual equivalence rather than changing the underlying construct being measured.

The instrument consisted of 12 items assessing various forms of controlling or demotivational teaching behaviors, including excessive criticism, intimidation, conditional regard, and controlling communication styles. Students evaluated the extent to which these behaviors were demonstrated by their PE teachers using a five-point Likert scale ranging from 1 (strongly disagree) to 5 (strongly agree). Higher scores indicated stronger perceptions of demotivational teaching practices. Sample items include: “My PE teacher pressures us to do things his or her way,” and “My PE teacher is less supportive when we do not meet expectations.” Previous research has demonstrated that the scale possesses strong reliability and construct validity in educational and sport contexts. In the present study, the scale demonstrated satisfactory internal consistency, with a Cronbach's alpha coefficient above 0.85.

#### Basic psychological needs

Students' basic psychological needs in physical education were assessed using the Basic Psychological Needs in Physical Education Scale (BPN-PE) developed by Ian G. Standage and colleagues. This instrument is grounded in Self-Determination Theory and is designed to measure the extent to which students experience satisfaction of the three fundamental psychological needs: autonomy, competence, and relatedness within the physical education environment. The scale consisted of 18 items distributed across three subscales: autonomy (6 items), competence (6 items), and relatedness (6 items).

Students responded to each item using a five-point Likert scale ranging from 1 (strongly disagree) to 5 (strongly agree). Higher scores indicated greater satisfaction of the respective psychological needs during physical education classes. Example items include: “*I feel that I have a say in how I participate in PE activities”* (autonomy), “*I feel capable of performing the exercises taught in PE”* (competence), and “*I feel connected with other students in my PE class”* (relatedness). The BPN-PE scale has been widely validated in previous research examining motivational processes in physical education settings. In the present study, the three subscales demonstrated good internal consistency, with Cronbach's alpha values ranging from 0.83 to 0.90, indicating high reliability.

It should be noted that the present study measured basic psychological need satisfaction rather than basic psychological need frustration. This distinction is important within Self-Determination Theory because need satisfaction and need frustration are related but conceptually distinct constructs. They should not be interpreted simply as opposite ends of a single continuum. Need satisfaction reflects the extent to which students feel autonomous, competent, and socially connected. In contrast, need frustration refers to the active obstruction or thwarting of these needs. For example, low autonomy satisfaction may indicate limited opportunities for choice, whereas autonomy frustration reflects feeling pressured, controlled, or forced to behave in a particular way. Because the present study focused only on need satisfaction, the statistical pathway linking demotivational teaching style and motivation may not fully capture the possible role of need frustration. Therefore, negative associations involving demotivational teaching style should be interpreted as associations with lower need satisfaction, not as direct evidence of active need frustration. This issue is acknowledged as a limitation and should be addressed in future studies by including both need satisfaction and need frustration measures. Simultaneously measuring need satisfaction and need frustration would allow future research to clarify whether demotivational teaching styles are associated with students' motivation through reduced satisfaction, increased frustration, or both processes.

#### Validity and reliability analysis

Prior to testing the structural relationships among the study variables, the reliability and validity of the measurement instruments were evaluated. Internal consistency reliability was first assessed using Cronbach's alpha coefficients, with values above 0.70 considered acceptable for research purposes. In addition, composite reliability (CR) was calculated to further evaluate the internal consistency of each construct, with recommended values exceeding 0.70. To examine the construct validity of the measurement model, a confirmatory factor analysis (CFA) was conducted using AMOS.

The CFA model was specified as a correlated multi-factor measurement model consisting of five latent constructs: motivational teaching style, demotivational teaching style, autonomy, competence, and relatedness. This latent structure was theoretically specified based on Self-Determination Theory, which distinguishes between perceived social-contextual teaching behaviors and students' basic psychological need satisfaction. Each observed item was allowed to load only on its theoretically assigned latent construct, and all cross-loadings were constrained to zero. Error terms were not freely correlated unless theoretically justified and supported by modification indices. No cross-loadings were permitted, and any consideration of error covariance was restricted to items within the same latent construct and only when conceptually defensible. Model respecification was not conducted solely on the basis of empirical modification indices. This approach was adopted to avoid overfitting the model to the present sample and to preserve the theoretical integrity of the measurement structure. This specification was used to ensure that the measurement model was consistent with the theoretical structure of Self-Determination Theory and the proposed study model.

The CFA was performed to verify whether the observed variables adequately represented the latent constructs of motivational teaching style, demotivational teaching style, and basic psychological need satisfaction, namely autonomy, competence, and relatedness. Model fit was evaluated using several widely recommended goodness-of-fit indices, including the chi-square to degrees of freedom ratio (χ^2^/df), comparative fit index (CFI), Tucker–Lewis index (TLI), root mean square error of approximation (RMSEA), and standardized root mean square residual (SRMR). For RMSEA, the 90% confidence interval was also reported to provide a more complete interpretation of model fit. Following established guidelines, acceptable model fit was indicated by χ^2^/df values below 3.0, CFI and TLI values above 0.90, and RMSEA and SRMR values below 0.08.

The measurement model demonstrated acceptable fit to the data: χ^2^/df = 2.14, CFI = 0.95, TLI = 0.94, RMSEA = 0.048, 90% CI (0.039, 0.056), and SRMR = 0.045. These indices indicated that the specified measurement model adequately represented the observed data. Convergent validity was evaluated using the average variance extracted (AVE) for each latent construct, with values >0.50 indicating that the construct explains more than half of the variance of its indicators. Standardized factor loadings were examined at the item level, and all retained items were expected to load substantially on their intended constructs. In the present study, standardized factor loadings ranged from 0.70 to 0.88, exceeding the recommended minimum threshold of 0.50. Detailed item-level factor loadings are reported in Table 1 to provide a more transparent evaluation of measurement quality. Reporting item-level standardized factor loadings allows readers to assess whether each observed indicator contributed adequately to its intended latent construct.

**Table 1:** Measurement model results.

Construct	Items	Factor loading	Cronbach's α	CR	AVE
Motivational teaching style	12	0.72–0.88	0.88	0.90	0.57
Demotivational teaching style	12	0.70–0.85	0.86	0.89	0.55
Autonomy	6	0.74–0.87	0.85	0.88	0.58
Competence	6	0.73–0.86	0.84	0.87	0.56
Relatedness	6	0.75–0.88	0.86	0.88	0.59

All constructs demonstrated satisfactory internal consistency (Cronbach's α >0.80), composite reliability above 0.85, and AVE above 0.50, supporting both convergent and discriminant validity.

Cronbach's alpha values ranged from 0.84 to 0.90, composite reliability values ranged from 0.87 to 0.91, and AVE values ranged from 0.55 to 0.59. These results supported the internal consistency and convergent validity of the constructs. More specifically, each construct demonstrated acceptable reliability, with Cronbach's alpha and CR values above 0.70, and adequate convergent validity, with AVE values above 0.50.

Discriminant validity was assessed using the Fornell–Larcker criterion by comparing the square root of AVE for each construct with its correlations with other constructs. This assessment was particularly important because the psychological need variables, autonomy, competence, and relatedness, are theoretically related and often empirically correlated within SDT-based research. The square root of AVE for each latent construct was greater than its correlations with other constructs, supporting discriminant validity based on the Fornell–Larcker criterion. Inter-construct correlations were also inspected to evaluate whether any constructs showed excessive overlap or potential redundancy. Although several constructs were moderately to strongly correlated, the results did not indicate that the constructs were empirically interchangeable. HTMT values were not estimated in the present study; therefore, discriminant validity was evaluated primarily using the Fornell–Larcker criterion and inter-construct correlations. Future studies should report HTMT values to provide additional evidence of discriminant validity.

To further address potential common method bias, a Harman's single-factor test was conducted. The results indicated that the first factor accounted for 32.46% of the total variance, which was below the commonly used 40% threshold, suggesting that common method variance was unlikely to pose a dominant threat to the validity of the findings. However, Harman's single-factor test was interpreted only as an initial diagnostic procedure, as this method alone is insufficient to fully rule out common method bias. Because all variables were collected using self-reported questionnaires from the same participants at a single point in time, the possibility of common method variance cannot be completely excluded. Therefore, the results should be interpreted with caution, and future studies should consider additional procedural and statistical remedies, such as multi-source data collection, temporal separation of measures, marker-variable techniques, or latent common method factor approaches.

Although the measurement model demonstrated acceptable global fit and satisfactory reliability and validity indices, several measurement-related limitations should be noted. First, the latent structure was tested within the present sample and should be further validated in different PE populations and cultural contexts. Second, all constructs were assessed using student self-report measures, which may introduce shared method variance and perception-based bias. Third, the present measurement model assessed need satisfaction but did not include need frustration, which limits the ability to distinguish lower need satisfaction from active psychological need thwarting. These considerations should be taken into account when interpreting the measurement and structural model results.

### Data analysis

Data analyses were conducted using IBM SPSS Statistics version 28.0 and AMOS version 28.0. Prior to the main analyses, the dataset was screened for missing values, outliers, patterned responses, and violations of normality assumptions. Missing data were examined during the initial data-screening stage. Responses were excluded when questionnaires contained substantial missing information, particularly when one or more main scale sections were incomplete or when missing responses prevented the computation of construct scores. Questionnaires with substantial incomplete responses were excluded from the analysis using listwise deletion, and no imputation was performed for excluded cases. Listwise deletion was considered appropriate because the excluded questionnaires did not provide sufficient valid information for the main study variables. Responses showing patterned answering were also removed before the final dataset was analyzed. Patterned answering was identified when respondents selected the same response option across most items, showed repetitive response sequences across different scale sections, or provided responses suggesting insufficient engagement with the questionnaire. Descriptive statistics, including means, standard deviations, skewness, and kurtosis values, were calculated for all study variables.

To examine the hypothesized associations among students' perceived motivational teaching style, students' perceived demotivational teaching style, and students' basic psychological need satisfaction, structural equation modeling (SEM) was employed using maximum likelihood estimation. SEM was chosen because it allows the simultaneous estimation of multiple relationships among latent constructs while accounting for measurement error. The analytical model was theoretically guided and explanatory in orientation, but it was not intended to establish causal or deterministic relationships among the variables.

The structural model was specified based on the theoretical framework of Self-Determination Theory, in which students' perceived motivational and demotivational teaching styles were hypothesized to be associated with students' satisfaction of autonomy, competence, and relatedness in physical education. Given the cross-sectional nature of the data, all structural paths were interpreted as theoretically specified associations rather than causal effects. The structural model was evaluated using several goodness-of-fit indices similar to those used in the measurement model evaluation. In addition, standardized path coefficients (β) and *p*-values were examined to determine the strength and significance of the hypothesized associations. The magnitude of standardized coefficients was also considered when interpreting the practical and theoretical relevance of the associations, rather than relying solely on statistical significance.

To test the potential mediating role of basic psychological needs, bootstrapping procedures with 5,000 resamples were applied to estimate the indirect associations. Direct, indirect, and total associations were reported to provide a more complete interpretation of the mediation model. The direct association referred to the pathway from perceived teaching style to students' motivational outcome after accounting for the mediators, whereas the indirect association referred to the pathway through autonomy, competence, and relatedness. The total association represented the combined direct and indirect associations. Mediation was interpreted as statistical mediation within a cross-sectional SEM framework, rather than as evidence of temporal or causal mediation. Mediation associations were considered significant when the 95% confidence interval did not include zero. Partial statistical mediation was inferred when both the direct and indirect associations remained significant. Statistical significance was set at *p* < 0.05 for all analyses.

Because all variables were measured using students' self-reported questionnaires at a single point in time, the possibility of common method bias was also considered. Several procedural precautions were used to reduce this risk, including anonymous questionnaire administration, assurances that there were no right or wrong answers, clarification that responses would not affect students' PE grades, and the use of separate questionnaire sections for different constructs. Harman's single-factor test was conducted as an initial diagnostic procedure; however, this approach was interpreted cautiously because it cannot fully rule out common method variance. Accordingly, common method bias was treated as a remaining methodological limitation rather than as an issue fully resolved by the statistical test.

Although the individual student was treated as the unit of analysis, students were nested within physical education classes and teachers. This hierarchical structure was not explicitly modeled in the present SEM analysis. Multilevel modeling was not applied because the present study focused on individual-level perceptual associations and because the number of higher-level units, particularly PE classes and teachers, was limited for estimating a stable multilevel SEM model. Therefore, the results should be interpreted with caution because potential classroom- or teacher-level dependency may have affected standard errors, confidence intervals, and significance tests. The estimated associations may therefore partly reflect shared classroom or teacher-level influences rather than purely individual-level perceptions. Future studies should consider multilevel SEM, hierarchical linear modeling, or cluster-robust standard errors to account for nested data structures. Longitudinal and multilevel research designs are also recommended to examine reciprocal and dynamic processes among perceived teaching behaviors, psychological need satisfaction, and students' autonomous motivational orientation over time.

### Conceptual framework

The present study is grounded in Self-Determination Theory (SDT), which posits that human motivation is closely associated with the satisfaction or frustration of three basic psychological needs: autonomy, competence, and relatedness. Within SDT, need satisfaction and need frustration are conceptually distinct processes rather than opposite ends of a single continuum. Low need satisfaction does not necessarily indicate active need frustration. In the context of physical education (PE), teaching behaviors play a central role in shaping these motivational processes. Motivational teaching styles, characterized by autonomy support, constructive feedback, and teacher involvement, are expected to be positively associated with the satisfaction of students' psychological needs. In contrast, demotivational teaching styles, including controlling behaviors, excessive criticism, and lack of student involvement, are likely to be negatively associated with these needs. Because the present study measured need satisfaction rather than need frustration, demotivational teaching styles are interpreted as being associated with lower need satisfaction, not as direct evidence of active need frustration.

**Figure 1:**
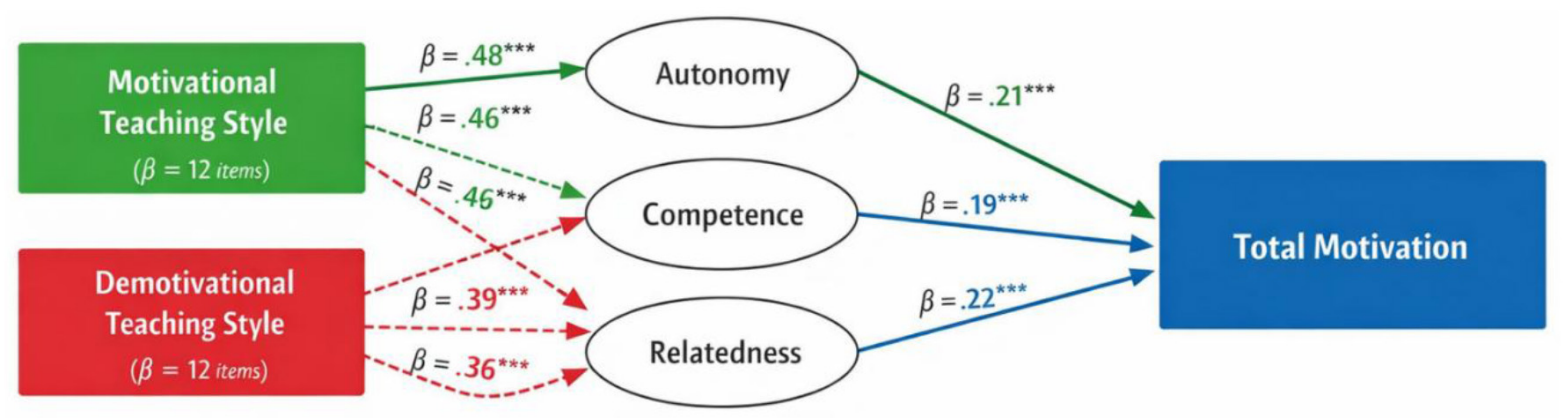
Conceptual model of motivational and demotivational teaching styles in physical education. ^***^p < 0.001.

Based on this theoretical framework, the study proposes that basic psychological need satisfaction functions as parallel statistical mediators between teaching styles and students' autonomous motivational orientation in PE. Figure 1 illustrates the conceptual model: motivational and demotivational teaching styles are hypothesized to be associated with the satisfaction of autonomy, competence, and relatedness, which in turn are associated with students' autonomous motivational orientation. However, this model is not intended to propose a fundamentally new SDT pathway. Rather, it provides a comparative extension of existing SDT-based research by examining motivational and demotivational teaching styles simultaneously within the same analytical model. The model is therefore theoretically guided and explanatory in orientation, but it should not be interpreted as a causal or deterministic framework because of the cross-sectional research design.

The contribution of this framework lies in comparing whether supportive and undermining instructional practices show similar or differentiated associations with students' basic psychological need satisfaction and autonomous motivational orientation. This comparative approach allows the simultaneous examination of supportive and undermining instructional practices without overstating the model as a novel or fully integrated theoretical framework. More specifically, the framework explores whether autonomy, competence, and relatedness show comparable or distinct mediating patterns across motivational and demotivational teaching styles. Thus, the study aims to provide additional comparative evidence regarding the psychological pathways through which perceived teaching styles are associated with students' motivational experiences in school-based PE. This contribution should be understood as an incremental refinement of established SDT-based PE research rather than a major theoretical advancement.

### Research hypotheses

Based on the conceptual framework, the study formulated the following hypotheses:

**H1:** Motivational teaching style positively predicts students' autonomy satisfaction in physical education.
**H2:** Motivational teaching style positively predicts students' competence satisfaction in physical education.
**H3:** Motivational teaching style positively predicts students' relatedness satisfaction in physical education.
**H4:** Demotivational teaching style negatively predicts students' autonomy, competence, and relatedness satisfaction in physical education.
**H5:** Basic psychological needs (autonomy, competence, and relatedness) mediate the relationship between motivational teaching style and students' motivation in physical education.
**H6:** Basic psychological needs (autonomy, competence, and relatedness) mediate the relationship between demotivational teaching style and students' motivation in physical education.

### Measurement model

To evaluate the measurement properties of the instruments, confirmatory factor analysis (CFA) was conducted. Table 1 summarizes the factor loadings, composite reliability (CR), and average variance extracted (AVE) for all constructs.

### Descriptive statistics and correlation

Table 2 presents the descriptive statistics and correlations among the study variables. These preliminary analyses provide evidence for the hypothesized relationships and help identify potential multicollinearity issues.

**Table 2:** Descriptive statistics and correlations.

Variable	Mean	SD	1	2	3	4	5	6
1. Motivational teaching style	4.12	0.56	–					
2. Demotivational teaching style	2.18	0.63	−0.41^**^	–				
3. Autonomy	4.03	0.59	0.52^**^	−0.47^**^	–			
4. Competence	3.98	0.61	0.49^**^	−0.44^**^	0.64^**^	–		
5. Relatedness	4.05	0.58	0.51^**^	−0.46^**^	0.61^**^	0.63^**^	–	
6. Total BPN	4.02	0.57	0.54^**^	−0.48^**^	0.91^**^	0.89^**^	0.90^**^	–

^**^p < 0.01.

These results indicate that motivational teaching style is positively correlated with all three psychological needs, while demotivational teaching style is negatively correlated, consistent with SDT predictions.

### Structural model results

The structural model was evaluated using structural equation modeling (SEM) in AMOS. The hypothesized model, in which motivational and demotivational teaching styles predicted students' basic psychological needs (autonomy, competence, relatedness), demonstrated a good overall fit to the data: χ^2^/df = 2.14, CFI = 0.95, TLI = 0.94, RMSEA = 0.048, SRMR = 0.045. These indices indicate that the measurement and structural models adequately represent the observed relationships. The path coefficients indicated that motivational teaching style had significant positive effects on all three psychological needs: autonomy (β = 0.48, *p* < 0.001), competence (β = 0.46, *p* < 0.001), and relatedness (β = 0.49, *p* < 0.001). In contrast, demotivational teaching style demonstrated significant negative effects on autonomy (β = −0.39, *p* < 0.001), competence (β = −0.36, *p* < 0.001), and relatedness (β = −0.38, *p* < 0.001). These results are consistent with the predictions of Self-Determination Theory, confirming that teacher behaviors have a strong impact on students' satisfaction of basic psychological needs.

### Mediation analysis

To examine the mediating role of basic psychological needs between teaching styles and students' overall motivation in physical education, a bootstrapping procedure with 5,000 resamples was conducted. The indirect effects and 95% bootstrap confidence intervals are presented in Table 3.

**Table 3:** Mediation analysis of basic psychological needs.

Predictor	Mediator	Outcome	Indirect effect (β)	95% CI	*p*-value	Significance
Motivational teaching style	Autonomy	Autonomous motivational orientation	0.21	0.15–0.28	< 0.001	Yes
Motivational teaching style	Competence	Autonomous motivational orientation	0.19	0.13–0.25	< 0.001	Yes
Motivational teaching style	Relatedness	Autonomous motivational orientation	0.22	0.16–0.28	< 0.001	Yes
Demotivational teaching style	Autonomy	Autonomous motivational orientation	−0.17	−0.23–0.11	< 0.001	Yes
Demotivational teaching style	Competence	Autonomous motivational orientation	−0.15	−0.21–0.10	< 0.001	Yes
Demotivational teaching style	Relatedness	Autonomous motivational orientation	−0.16	−0.22–0.10	< 0.001	Yes

Autonomous Motivational Orientation refers to the composite score of intrinsic motivation and identified regulation in PE, both of which represent autonomous forms of motivation within SDT. It should not be interpreted as total motivation across all SDT regulations. Bootstrap confidence intervals excluding zero indicate significant mediation effects.

These results indicate that basic psychological needs significantly mediate the relationship between both motivational and demotivational teaching styles and students' motivation, providing empirical support for the SDT-based mechanism proposed in the conceptual model. In other words, teacher behaviors influence students' motivation indirectly through the satisfaction or frustration of autonomy, competence, and relatedness.

### Summary of findings

1. Motivational teaching style positively predicts autonomy, competence, and relatedness, which in turn enhances student motivation.
2. Demotivational teaching style negatively predicts the same needs, reducing student motivation.
3. Mediation analysis confirms that basic psychological needs fully or partially mediate these relationships, highlighting the central role of psychological needs in the PE motivational process.

These findings not only support the hypotheses (H1–H6) but also provide a comprehensive SDT-based model linking teaching styles to students' motivational outcomes in physical education.

## Results

### Descriptive statistics and correlations

Table 4 presents the descriptive statistics, skewness, kurtosis, and intercorrelations among the study variables. Participants reported relatively high perceptions of motivational teaching style (*M* = 4.12, SD = 0.56) and moderate perceptions of demotivational teaching style (*M* = 2.18, SD = 0.63). The basic psychological needs of autonomy, competence, and relatedness were generally rated positively (*M* range = 3.98–4.05, SD range = 0.58–0.61), indicating moderate to high levels of need satisfaction during physical education (PE) classes. Skewness and kurtosis values were within the acceptable range (±1), suggesting that the data were approximately normally distributed. Minimum and maximum values also indicated sufficient variability for further analysis.

**Table 4:** Descriptive statistics, skewness, kurtosis, and correlations.

Variable	*M*	SD	Min	Max	Skewness	Kurtosis	1	2	3	4	5
1. motivational teaching Style	4.12	0.56	2.50	5.00	−0.45	0.28	–				
2. Demotivational teaching style	2.18	0.63	1.00	4.50	0.52	−0.12	−0.41^**^	–			
3. Autonomy	4.03	0.59	2.33	5.00	−0.38	0.33	0.52^**^	−0.47^**^	–		
4. Competence	3.98	0.61	2.00	5.00	−0.31	0.41	0.49^**^	−0.44^**^	0.64^**^	–	
5. Relatedness	4.05	0.58	2.50	5.00	−0.29	0.35	0.51^**^	−0.46^**^	0.61^**^	0.63^**^	–

^**^p < 0.01.

**Figure 2:**
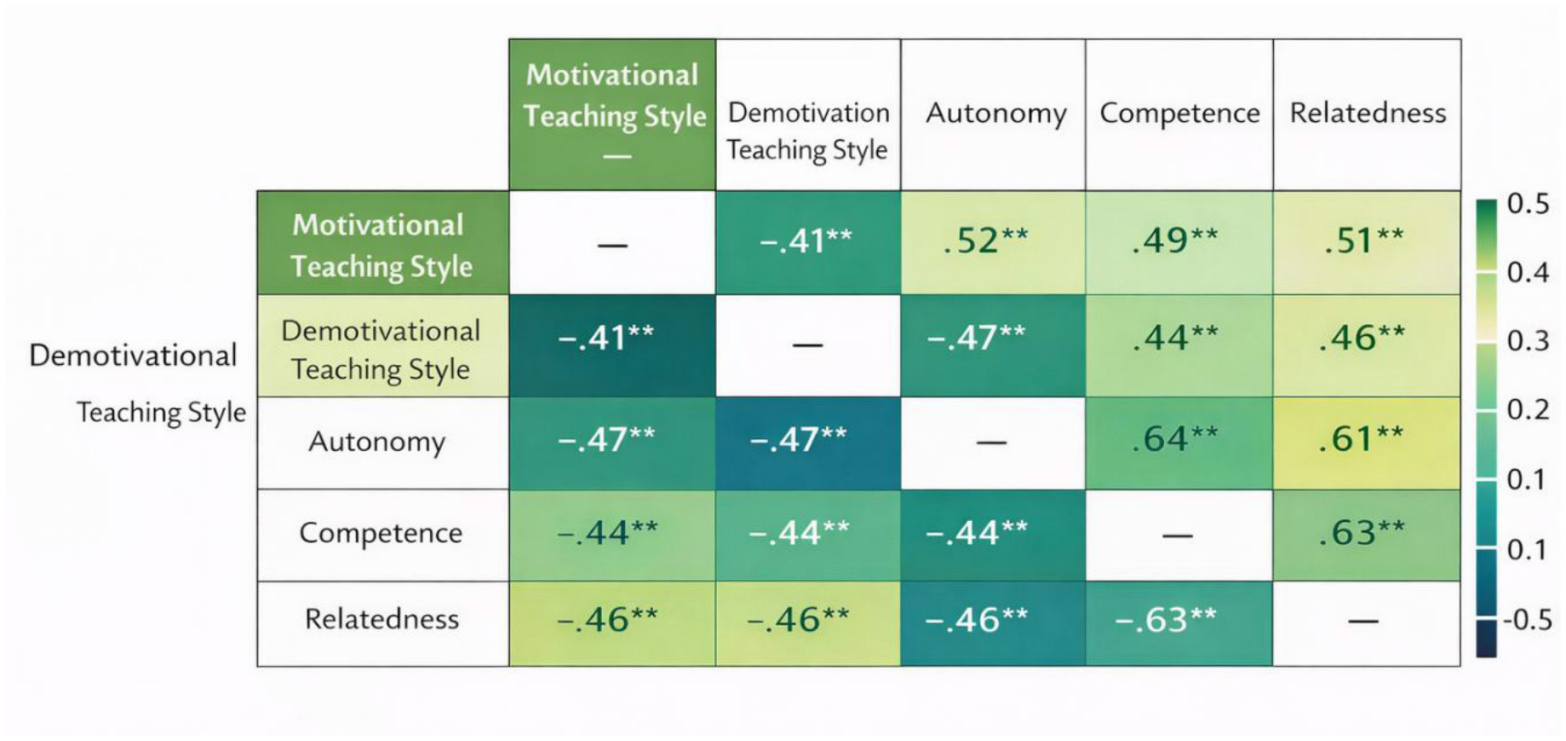
Heatmap of correlation among study variables. ^**^p < 0.01.

Correlation analyses showed that perceived motivational teaching style was positively correlated with autonomy, competence, and relatedness, whereas perceived demotivational teaching style was negatively correlated with all three psychological needs. Figure 2 visually summarizes these results through a heatmap, showing the direction and relative strength of the associations. The heatmap also illustrates positive intercorrelations among autonomy, competence, and relatedness, indicating that students with higher satisfaction of one psychological need tended to report higher satisfaction of the other needs.

Although the correlations among autonomy, competence, and relatedness were moderate to strong, they did not indicate that the constructs were interchangeable. Therefore, discriminant validity was further evaluated in the measurement model using AVE-based criteria and other validity indicators, as reported in the measurement model results. The correlational results provided preliminary support for the hypothesized associations among perceived teaching styles and students' basic psychological need satisfaction. Because these results are based on cross-sectional correlations, they should not be interpreted as evidence of causal effects. Multicollinearity diagnostics were also examined. Variance inflation factor (VIF) values were below the commonly accepted threshold of 3.0, and tolerance values were above the recommended minimum threshold, indicating that multicollinearity among predictors was not a serious concern. Overall, the descriptive and correlational findings supported the suitability of the data for testing the hypothesized structural model.

### Measurement model

The measurement model was assessed using confirmatory factor analysis (CFA). Factor loadings for all items ranged from 0.70 to 0.88, indicating that the observed variables adequately represented their latent constructs. The composite reliability (CR) values for all constructs exceeded 0.85, and the average variance extracted (AVE) values ranged from 0.55 to 0.59, supporting convergent validity. Because several constructs were moderately to strongly correlated, discriminant validity was examined carefully by comparing the square root of AVE with the inter-construct correlations. The results indicated that each construct remained empirically distinguishable from the others. This assessment was particularly relevant for autonomy, competence, and relatedness, as strong correlations among psychological need variables may raise concerns about their empirical distinctiveness.

Harman's single-factor test showed that the first factor accounted for < 40% of the total variance, suggesting that common method bias was unlikely to dominate the results. However, this finding should be interpreted cautiously because Harman's single-factor test is only an initial diagnostic procedure and cannot fully rule out common method variance. Overall, the CFA results provided satisfactory evidence of reliability, convergent validity, and discriminant validity for the subsequent SEM analysis.

### Structural model

The hypothesized structural model was evaluated using structural equation modeling (SEM) in AMOS. The model demonstrated good fit to the data, with χ^2^(142) = 304.12, *p* < 0.001, χ^2^/df = 2.14, CFI = 0.95, TLI = 0.94, RMSEA = 0.048 [90% CI (0.039, 0.056)], and SRMR = 0.045. These fit indices indicated that the proposed associations among perceived motivational and demotivational teaching styles, basic psychological need satisfaction, and students' autonomous motivational orientation were adequately represented.

As shown in Table 5, perceived motivational teaching style was positively associated with autonomy, competence, and relatedness, whereas perceived demotivational teaching style was negatively associated with all three psychological needs. The standardized coefficients were moderate to large for motivational teaching style and moderate for demotivational teaching style, indicating meaningful associations in the expected directions. Among the positive paths, the strongest association was observed for relatedness, suggesting that students' sense of social connection may be particularly salient in relation to perceived motivational teaching behaviors. For perceived demotivational teaching style, the strongest negative association was observed for autonomy, suggesting that students' sense of agency may be especially sensitive to perceived controlling or demotivational instructional behaviors.

**Table 5:** Structural model and mediation analysis results.

Predictor	Mediator	Outcome	Path type	β	SE	*p*-value	95% CI	Significance
Motivational teaching style (MTS)	–	Autonomy	Direct	0.48	0.05	< 0.001	–	Yes
Motivational teaching style (MTS)	–	Competence	Direct	0.46	0.05	< 0.001	–	Yes
Motivational teaching style (MTS)	–	Relatedness	Direct	0.49	0.05	< 0.001	–	Yes
Demotivational teaching style (DTS)	–	Autonomy	Direct	−0.39	0.06	< 0.001	–	Yes
Demotivational teaching style (DTS)	–	Competence	Direct	−0.36	0.06	< 0.001	–	Yes
Demotivational teaching style (DTS)	–	Relatedness	Direct	−0.38	0.06	< 0.001	–	Yes
Motivational teaching style (MTS)	Autonomy	Autonomous motivational orientation	Indirect	0.21	0.04	< 0.001	0.15–0.28	Yes
Motivational teaching style (MTS)	Competence	Autonomous motivational orientation	Indirect	0.19	0.04	< 0.001	0.13–0.25	Yes
Motivational teaching style (MTS)	Relatedness	Autonomous motivational orientation	Indirect	0.22	0.04	< 0.001	0.16–0.28	Yes
Demotivational teaching style (DTS)	Autonomy	Autonomous motivational orientation	Indirect	−0.17	0.04	< 0.001	−0.23 to −0.11	Yes
Demotivational teaching style (DTS)	Competence	Autonomous motivational orientation	Indirect	−0.15	0.04	< 0.001	−0.21 to −0.10	Yes
Demotivational teaching style (DTS)	Relatedness	Autonomous motivational orientation	Indirect	−0.16	0.04	< 0.001	−0.22 to −0.10	Yes

Because the present study measured need satisfaction rather than need frustration, the negative paths should be interpreted as associations with lower need satisfaction, not as direct evidence of active need frustration. Mediation analyses using bootstrapping with 5,000 resamples indicated that autonomy, competence, and relatedness statistically mediated the associations between teaching styles and students' autonomous motivational orientation. As presented in Table 5, the indirect associations were significant for both perceived motivational and demotivational teaching styles, as the 95% confidence intervals did not include zero. Relatedness showed the strongest indirect association for perceived motivational teaching style, whereas autonomy showed the strongest indirect association for perceived demotivational teaching style. Overall, the indirect associations were small to moderate in magnitude, suggesting that psychological need satisfaction accounted for a meaningful but not exclusive portion of the associations between perceived teaching styles and autonomous motivational orientation.

These mediation results should be understood as statistical pathways within a cross-sectional SEM model rather than evidence of causal or temporal mechanisms. The present model treated autonomy, competence, and relatedness as parallel mediators; therefore, it did not examine whether these needs operate sequentially, interactively, or as a combined higher-order need satisfaction construct. Covariances among residuals of the three needs were moderate (*r* = 0.32–0.41, *p* < 0.001), consistent with the conceptual expectation that the needs are interrelated but distinct. Variance inflation factors for all predictors were below 3.0, indicating no serious multicollinearity concerns.

### Mediation analysis

The mediating role of basic psychological needs (autonomy, competence, and relatedness) in the associations between teaching styles and students' autonomous motivational orientation was examined using bootstrapping with 5,000 resamples in AMOS. As shown in Table 6, the indirect associations between motivational teaching style (MTS) and autonomous motivational orientation through autonomy, competence, and relatedness were statistically significant, as the 95% confidence intervals did not include zero. The direct association between MTS and autonomous motivational orientation also remained significant, indicating partial statistical mediation. Among the three mediators, relatedness showed the strongest indirect association for MTS.

**Table 6:** Direct, indirect, and total associations in the mediation model.

Predictor	Mediator	Outcome	Effect type	β	SE	*p*-value	95% CI	Significance
Motivational teaching style (MTS)	–	Autonomous motivational orientation	Direct	0.27	0.05	< 0.001	–	Yes
MTS	Autonomy	Autonomous motivational orientation	Indirect	0.21	0.04	< 0.001	0.15 – 0.28	Yes
MTS	Competence	Autonomous motivational orientation	Indirect	0.19	0.04	< 0.001	0.13 – 0.25	Yes
MTS	Relatedness	Autonomous motivational orientation	Indirect	0.22	0.04	< 0.001	0.16 – 0.28	Yes
MTS	–	Autonomous motivational orientation	Total Effect	0.89	0.05	< 0.001	0.79 – 0.98	Yes
Demotivational Teaching Style (DTS)	–	Autonomous motivational orientation	Direct	−0.21	0.05	< 0.001	–	Yes
DTS	Autonomy	Autonomous motivational orientation	Indirect	−0.17	0.04	< 0.001	−0.23 to −0.11	Yes
DTS	Competence	Autonomous motivational orientation	Indirect	−0.15	0.04	< 0.001	−0.21 to −0.10	Yes
DTS	Relatedness	Autonomous motivational orientation	Indirect	−0.16	0.04	< 0.001	−0.22 to −0.10	Yes
DTS	–	Autonomous motivational orientation	Total Effect	−0.69	0.05	< 0.001	−0.79 to −0.60	Yes

For demotivational teaching style (DTS), the indirect associations through autonomy, competence, and relatedness were also statistically significant. The direct association between DTS and autonomous motivational orientation remained significant, indicating partial statistical mediation. Among the three psychological needs, autonomy showed the strongest indirect association for DTS. These results suggest that the indirect pathways differed in relative magnitude across supportive and demotivational teaching styles.

The mediation model should be understood as a parallel statistical mediation model. Autonomy, competence, and relatedness were examined as simultaneous mediators rather than sequential or serial mediators. Therefore, the findings do not determine whether one psychological need precedes or strengthens another over time. Given the cross-sectional design, the mediation results should not be interpreted as evidence of causal or temporal mechanisms. In addition, because only need satisfaction was measured, the negative indirect associations involving DTS should be understood as pathways through lower need satisfaction rather than direct evidence of need frustration.

## Discussion

The present study examined how perceived motivational and demotivational teaching styles were associated with students' basic psychological need satisfaction and autonomous motivational orientation in physical education (PE). Given the cross-sectional design, the findings are best interpreted as theoretically meaningful associations rather than evidence of causal effects. Overall, the findings are broadly consistent with Self-Determination Theory (SDT), which emphasizes the role of social-contextual conditions in students' psychological need satisfaction and motivational quality (Lee et al., 2021; Ryan and Deci, 2000). Rather than being interpreted as evidence of a new SDT pathway, the results should be viewed as a comparative extension of established SDT assumptions within the specific context of school-based PE.

The positive associations between motivational teaching style and autonomy, competence, and relatedness are consistent with prior studies in educational and sport settings (Tilga et al., 2021). However, beyond merely confirming previous SDT-based findings, the present results may be interpreted in relation to the distinctive pedagogical characteristics of PE. Unlike many classroom subjects, PE involves visible bodily performance, peer interaction, skill comparison, public feedback, and frequent teacher–student and student–student interaction. In such a context, students' motivational experiences may be particularly sensitive to how teachers communicate expectations, structure tasks, provide choices, and respond to mistakes. Autonomy-supportive behaviors, such as offering meaningful choices, encouraging self-initiation, and providing constructive feedback, may help students feel more agentic, competent, and socially accepted. This interpretation is consistent with SDT, which emphasizes that social-contextual support is closely related to students' basic psychological need satisfaction and motivational quality (Haerens et al., 2015; Ryan and Deci, 2000). At the same time, this association may also reflect students' prior motivational orientation. Students who already enjoy PE or perceive themselves as competent may be more likely to interpret teacher behaviors as supportive, even when similar instructional practices are experienced differently by less motivated students.

A particularly important finding is that relatedness appeared to be the strongest statistical pathway linking perceived motivational teaching style and students' motivation. This may be explained by the inherently social nature of PE, where learning often occurs through team activities, peer cooperation, group practice, and public performance. In this environment, motivational teaching may not only support students' autonomy and competence but also strengthen their sense of belonging and interpersonal safety. When teachers create an inclusive and respectful classroom climate, students may be more willing to participate, take risks, and remain engaged even when physical tasks are challenging. This interpretation is supported by previous research showing that teacher and peer support are important sources of relatedness, motivation, and affective responses in PE (Cox et al., 2015; Standage et al., 2003). It also suggests that relatedness should not be treated as a secondary need in PE contexts, because students' willingness to engage may depend strongly on whether they feel accepted and supported within the learning environment. Nevertheless, this interpretation should be treated with caution. In some PE contexts, competence may be more central than relatedness, particularly when lessons emphasize skill acquisition, performance assessment, or physical ability comparison. Therefore, the relative importance of autonomy, competence, and relatedness may vary depending on instructional content, assessment practices, peer climate, and students' prior movement experiences.

Conversely, perceived demotivational teaching style, characterized by controlling behaviors, excessive criticism, psychological pressure, and limited student involvement, was negatively associated with all three psychological needs. This result is consistent with previous studies showing that controlling or need-thwarting teaching behaviors are linked to lower motivation and less favorable psychological outcomes in PE (Haerens et al., 2015; Opdenakker, 2021; Viksi and Tilga, 2022). The strongest negative indirect pathway for demotivational teaching style was observed through autonomy. This is theoretically plausible because controlling practices may directly constrain students' sense of agency, choice, and self-regulation. In PE, where students are often publicly evaluated through physical performance, controlling language, public criticism, intimidation, or excessive pressure may be especially harmful to students' perceived autonomy and emotional safety. However, an alternative interpretation is also possible. Students with lower perceived competence or negative prior experiences in PE may be more sensitive to teacher criticism and may therefore report stronger perceptions of demotivational teaching. This possibility reinforces the need to interpret the present findings as associations rather than directional effects.

These findings also support the argument that motivational and demotivational teaching styles should not be interpreted simply as opposite poles of a single continuum. Supportive teaching may primarily create conditions for need satisfaction, whereas controlling or demotivational teaching may actively contribute to need frustration. These processes may not be symmetrical. For example, supportive feedback may strengthen confidence and engagement, but public criticism or pressure may have a disproportionately stronger negative association with autonomy and perceived safety. This interpretation is consistent with research distinguishing autonomy-supportive and controlling teaching as potentially unique motivational pathways rather than simple opposites (Haerens et al., 2015; Hanton et al., 2013). Therefore, the present study contributes to the literature by examining both supportive and undermining instructional styles within a single model and by showing that they may be differentially associated with students' psychological needs. However, because the present study measured need satisfaction rather than need frustration, the negative pathways involving demotivational teaching style should be interpreted as associations with lower need satisfaction, not as direct evidence of active need frustration. Future research should test both need satisfaction and need frustration simultaneously to clarify whether demotivational teaching styles are linked to motivation through distinct frustration-based mechanisms.

Although SDT provides the main theoretical framework for interpreting these findings, alternative perspectives may also help explain the observed patterns. From the perspective of Achievement Goal Theory, motivational teaching may be associated with a mastery-oriented climate in which effort, progress, and learning are emphasized. In contrast, demotivational teaching may reflect a more performance-pressured climate characterized by comparison, evaluation, and fear of failure (Bandura, 1977; Standage et al., 2003). Similarly, Social Cognitive Theory suggests that constructive feedback and progressive task challenges may be linked to stronger self-efficacy, whereas repeated criticism may weaken students' confidence in their physical abilities (Bandura, 1977; Podsakoff et al., 2003). These complementary perspectives indicate that the associations observed in this study may reflect not only need satisfaction processes, but also broader motivational mechanisms involving mastery climate, self-efficacy, perceived competence, and task value. Expectancy-Value Theory may also offer a complementary explanation. Students may be more motivated in PE when they perceive activities as valuable, useful, and personally relevant, regardless of whether motivation is primarily explained through autonomy, competence, or relatedness. From this perspective, teaching practices that clarify the purpose and relevance of PE tasks may be associated with students' motivation through perceived value as well as through psychological need satisfaction. Taken together, these perspectives suggest that SDT provides an important but not exhaustive explanation of students' motivation in PE. Motivation may also depend on achievement climate, efficacy beliefs, perceived task value, peer norms, and broader classroom ecology.

The mediation findings should be understood as theoretically specified statistical pathways rather than temporal mechanisms. This distinction is important because students' perceptions of teaching styles, psychological need satisfaction, and motivational orientation may be reciprocally related. Students who are already more motivated may evaluate their teachers more positively, whereas less motivated students may perceive similar instructional behaviors as more controlling or discouraging. Similarly, students with stronger peer relationships or higher prior competence in PE may report both greater need satisfaction and more favorable perceptions of teaching style. Classroom-level factors, such as peer norms, class climate, teacher experience, and school culture, may also shape these associations. Because the present analysis did not model students nested within classes and teachers, these contextual influences may not have been fully separated from individual-level patterns.

The use of a composite motivation outcome also requires theoretical caution. Within SDT, motivation is multidimensional, and different forms of motivation reflect different degrees of self-determination, including intrinsic motivation, identified regulation, introjected regulation, external regulation, and amotivation (Jakobsen and Jensen, 2015; Ryan and Deci, 2000; Siacor et al., 2024). Therefore, the motivation outcome in this study should be understood as an overall indicator of students' adaptive motivational orientation in PE rather than as a comprehensive representation of all motivational regulations. Future studies should examine autonomous motivation, controlled motivation, and amotivation separately to determine whether the observed pathways differ across specific motivational regulations. This distinction is important because supportive teaching may be more strongly associated with autonomous motivation, whereas demotivational teaching may be more closely linked to controlled motivation or amotivation. For example, autonomy-supportive teaching may be more closely related to intrinsic enjoyment, whereas clear explanations of task value may be more closely related to identified regulation. Examining these components separately would allow future studies to identify whether different teaching behaviors are associated with different forms of autonomous motivation.

From a practical perspective, the findings underscore the importance of encouraging PE teachers to adopt autonomy-supportive instructional strategies while minimizing controlling practices. Rather than providing only general encouragement, teacher training programs should include concrete classroom strategies that support students' autonomy, competence, and relatedness. Specific recommendations include offering meaningful choices, allowing students to select task difficulty levels, inviting them to set personal performance goals, and explaining the rationale behind learning tasks. To support competence, teachers should provide constructive feedback, use progressive task challenges, emphasize individual improvement, and avoid excessive comparison among students. To strengthen relatedness, teachers should foster a socially inclusive class environment through cooperative learning, supportive peer interaction, inclusive grouping, and respectful teacher–student communication. At the same time, PE teachers should reduce demotivational practices, such as public criticism, controlling language, excessive pressure, intimidation, and punitive responses to mistakes. These recommendations should be interpreted in light of the broader classroom context. In some PE settings, structured instruction and clear behavioral expectations may be necessary for safety, organization, and effective skill learning. Therefore, the goal is not to eliminate structure, but to provide structure in ways that support autonomy, competence, and relatedness rather than relying on pressure or controlling communication.

Despite its contributions, the study has several limitations. First, the cross-sectional design precludes causal inference. Accordingly, the observed relationships should be interpreted as associations rather than causal pathways. Although the SEM results are consistent with theoretically plausible directional relationships, the present design cannot determine whether perceived teaching styles precede changes in students' psychological need satisfaction and autonomous motivational orientation. Reciprocal or dynamic processes may also exist. For example, students with higher motivation may perceive teacher behaviors more positively, whereas less motivated students may interpret similar instructional behaviors as more controlling or discouraging. Teachers may also adapt their instructional practices in response to students' engagement, effort, or classroom behavior. These possibilities should be examined in future longitudinal or intervention-based studies.

Second, the sample was drawn from secondary schools in a specific region, which may limit generalizability to other educational or cultural contexts. Because PE curricula, teacher–student interaction norms, and motivational climates may vary across schools, regions, and cultures, future research should test the model in more diverse educational settings and with larger multi-school samples.

Third, the hierarchical structure of the data was not explicitly modeled. Students were nested within PE classes and teachers, which may have introduced dependency among responses from students sharing the same instructional environment. Because conventional SEM assumes independent observations, the absence of multilevel modeling or cluster-adjusted standard errors may have affected the precision of parameter estimates, standard errors, confidence intervals, and significance tests. Future studies should consider multilevel SEM, hierarchical linear modeling, or cluster-robust analytical approaches to account for classroom- and teacher-level effects.

Fourth, all measures relied on students' self-reported perceptions. In particular, teaching styles were measured through students' perceptions rather than objectively observed teacher behaviors. Students' evaluations may have been influenced by personal preferences, prior PE experiences, general attitudes toward the teacher, classroom satisfaction, or social desirability. This subjective measurement approach may have affected the strength of the observed associations among teaching styles, psychological need satisfaction, and autonomous motivational orientation. Future research should incorporate additional data sources, such as teacher self-reports, peer evaluations, independent observer ratings, and systematic classroom observations.

A further limitation concerns the potential risk of common method bias. Although Harman's single-factor test suggested that the data were not dominated by a single factor, this procedure alone cannot fully rule out common method variance. Because all variables were collected from the same participants using the same self-report method at a single time point, the observed associations may have been influenced by shared method variance, response tendencies, or students' general perceptions of their PE teacher and classroom environment. Future studies should adopt stronger procedural and statistical remedies, including multi-source data collection, temporal separation of measures, marker-variable techniques, or latent common method factor approaches.

Another measurement-related limitation is that the present study assessed basic psychological need satisfaction but did not measure basic psychological need frustration. This issue is particularly relevant to the interpretation of demotivational teaching styles. Within Self-Determination Theory, need satisfaction and need frustration are related but conceptually distinct constructs. Lower need satisfaction does not necessarily indicate active need frustration. Demotivational or controlling teaching behaviors may be associated not only with reduced satisfaction of autonomy, competence, and relatedness, but also with direct experiences of autonomy frustration, competence frustration, and relatedness frustration. Therefore, the psychological mechanisms underlying demotivational teaching styles may not have been fully captured in the present model. Future research should include both need satisfaction and need frustration measures to provide a more comprehensive understanding of how perceived demotivational teaching styles are linked to students' motivational experiences in PE.

Finally, the operationalization of the motivation outcome requires caution. The present study used autonomous motivational orientation as a composite indicator based on intrinsic motivation and identified regulation. Although both regulations represent autonomous forms of motivation within SDT, combining them may obscure differences between enjoyment-based and value-based motivation. Future studies should examine intrinsic motivation, identified regulation, controlled motivation, and amotivation separately to provide a more differentiated understanding of motivational processes in PE. Moderating factors such as student age, gender, prior physical activity experience, and classroom climate should also be considered in future research.

## Conclusion

This study provides evidence that perceived motivational and demotivational teaching styles are meaningfully associated with students' basic psychological need satisfaction and autonomous motivational orientation in physical education. Autonomy, competence, and relatedness functioned as statistical mediating pathways, indicating that students' psychological need experiences are central to understanding how perceived teaching behaviors are linked to motivation in PE. Rather than proposing a new theoretical pathway, the study offers a comparative extension of SDT-based PE research by examining supportive and controlling instructional behaviors within the same analytical model. From a practical standpoint, the findings highlight the importance of teacher training programs that promote autonomy-supportive strategies, constructive feedback, and inclusive interaction while reducing controlling practices. Given the cross-sectional design, reliance on self-reported perceptions, and focus on need satisfaction rather than need frustration, the findings should be interpreted as associational rather than causal. Future research should extend these findings through longitudinal designs, cross-cultural comparisons, multilevel analyses, and intervention studies to strengthen causal inference and generalizability.

## Data Availability

The original contributions presented in the study are included in the article/supplementary material, further inquiries can be directed to the corresponding author.
